# Multiple chemical sensitivity caused by exposure to ignition coal fumes: a case report

**DOI:** 10.1186/2052-4374-25-32

**Published:** 2013-11-01

**Authors:** Myeong-Ja Yun, Dong-Mug Kang, Kyoung-Hye Lee, Young-Ki Kim, Jong-Eun Kim

**Affiliations:** 1 Department of Preventive and Occupational Medicine, School of Medicine, Pusan National University, Pusan, South Korea; 2 Department of Occupational & Environmental Medicine, Pusan National University Yangsan Hospital, Yangsan, South Korea

**Keywords:** Multiple chemical sensitivity, Quick environmental exposure and sensitivity inventory, Ignition coal, Combustion product, Fibromyalgia syndrome

## Abstract

**Background:**

Although multiple chemical sensitivity (MCS) is a well-known disorder caused by environmental exposures, MCS caused by occupational exposure has been reported in Korea. Therefore, we report a MCS case caused by environmental exposure to ignition coal after a differential diagnosis to exclude other diseases.

**Case report:**

Since 2011, a 55-year-old woman had experienced edema, myalgia, and other symptoms when she smelled ignition coal near her workplace. She had been diagnosed with fibromyalgia syndrome(FMS) and was treated, with no improvement of symptoms. Since then, she showed the same symptoms after exposure to city gas, the smell of burning, and exhaust gas. To avoid triggering substances, she moved to a new house and used an air purifier. She quit her job in November 2012. After visiting our hospital, she underwent a differential diagnosis for FMS, chronic fatigue syndrome, and somatization disorder. She was diagnosed with MCS by the Korean version of the Quick Environment Exposure Sensitivity Inventory (QEESI). She was educated about the disease and to avoid triggering substances. She received ongoing treatment for her symptoms.

**Conclusion:**

This case showed that symptoms began after smelling ignition coal. After that, her triggers was increased such as the smell of city gas, burning, and exhaust gas. This case is the first reported in Korea of MCS due to environmental exposure after ruling out other diseases.

## Background

Multiple chemical sensitivity (MCS) is a disorder caused by exposure to extremely low levels of chemicals, which exhibits various physical and psychological symptoms [1]. MCS is not a well-established disease in the medical community, it has no confirmatory diagnostic criteria. The definition of MCS (also referred to as toxicant-induced loss of tolerance (TILT), idiopathic environmental intolerance (IEI), ecologic illness, allergic toxemia, or immune system dysregulation) has changed over time because the symptoms are ambiguous and difficult to diagnose. In 1996, the World Health Organization (WHO) expanded the concept of MCS into the disease which is unexplainable by certain physical and physiological diseases, and which is recurrent disorder affected by usually unrecognizable environmental chemicals [2]. Cullen proposed diagnostic criteria for MCS in 1987: symptoms that could cause harm by an exposure level less than one lower standard deviation below the average [1]. Since then, diagnostic criteria for MCS were agreed upon in Atlanta [3] in 1999, but there is no confirmatory diagnostic test.

The prevalence of MCS has been reported in various studies [4,5]. MCS shows various non-specific symptoms such as headache, concentration deficiency, fatigue, pain, skin lesions, and nausea. Sensitivity for a certain chemical can expand to sensitivity to other chemicals [6]. Patients have shown a variety of changing behaviors, including avoiding triggering substances and wearing a mask, moving to a new house, and quitting a job after the onset of symptoms [7]. Causative agents of MCS are usual materials easily found in everyday life such as household cleaning agents, smoking, car exhaust, new construction or repair of buildings, air pollutants, food, and electromagnetic waves [8]. If MCS is suspected, the Quick Environmental Exposure and Sensitivity Inventory (QEESI), developed in 1996 by Miller and Prihoda, can be used as a screening test [9]. MCS may co-occur with allergic diseases. However, allergies alone cannot explain all of the symptoms. The diagnosis of MCS should involve ruling out other similar diseases such as somatization disorder, chronic fatigue syndrome (CFS), and fibromyalgia (FMS). However, sometimes MCS can occur with other diseases [10].

Few studies about MCS in Korea have been published (Table 1). Previous studies on volatile organic chemicals (VOCs) among construction workers [11] and white-collar workers [12] were cross-sectional studies using a self-reported symptom questionnaire. Epidemiological studies on MCS in Korea have included one on the prevalence of self-reported MCS in a community-based sample survey [13] and one on the prevalence of self-reported MCS among Hebei Spirit oil spill clean-up workers [14]. In these epidemiological studies that used the Korean version of the QEESI, no differential diagnostic process was performed. A case report of chemical analysis department workers [15] presented as a conference poster used the Korean version of the QEESI and conducted a differential diagnosis through a clinical diagnostic process. However, this report is a case of occupational exposure and has not been published yet. Therefore, MCS has been reported only in conjunction with occupational exposure in Korea. No report has been previously published in Korea on MCS from environmental substances to which people could be exposed in everyday life. In this report, were report a case of MCS caused by ignition coal and combustion products (such as city gas) with a clinical diagnosis, ruling out other diseases and a literature review.

**Table 1:** Papers about multiple chemical sensitivity in Korea

**Reference**	**Year**	**Form of publication**	**Study subjects**	**Diagnostic criteria**
Kim EJ [11].	2004	Master’s thesis	Construction workers	Symptom self-report
Sung KC [12].	2005	Master’s thesis	Office workers	Symptom self-report
Jeon BH et al [13].	2012	Poster presentation	Community-based sample survey	Korean version of QEESI^*^
Jeon BH et al [14].	2012	Poster presentation	Hebei Spirit oil spill clean-up workers	Korean version of QEESI^*^
Lee DH et al [15].	2008	Case presentation	Chemical analysis department workers	Doctor-diagnosed MCS^†^

^*^QEESI, Quick Environmental Exposure and Sensitivity Inventory.

^†^MCS, multiple chemical sensitivity.

## Case presentation

### Patient information

A 55-year-old female.

### Chief complaint

Swelling in the body after exposure to ignition coal fumes.

### Present illness

She had operated a second-floor bar 5 years. In 2011, she experienced body swelling, sputum, and muscular symptoms beginning when the meat roasting restaurant on the first floor of the same building replaced charcoal with ignition coal a few weeks before presentation to the division of rheumatology. She was diagnosed with FMS and treated. Her symptoms were improved when she took her prescribed FMS medicine. Her symptoms recurred after exposure to the fumes of ignition coal when she returned to work. Within a few weeks, she moved into a new house which used city gas (previously, she had lived in a house using an oil boiler). Her symptoms, such as whole body swelling (both arms and both calves were especially edematous), sputum, myalgia, and arthralgia worsened, and she had the new onset of headache after exposure to the smell of city gas. She vomited at night when she moved to a new house. To remove the city gas fumes, she began to use an air purifier. If symptoms recurred, she took FMS medication, but the symptoms did not improve. Eventually, 1 year later, she moved into a different house. Gradually, she developed a sensitivity to the smell of burning and exhaust gas. After exposure to the fumes, symptoms such as swelling, myalgia, arthralgia, poor concentration, and nausea occurred. The symptoms consistently occurred when she inhaled the fumes of ignition coal, so she finally quit her job 1 year later from first induction of symptoms. She underwent a pulmonary function test because she had difficulty breathing, whole body swelling, and a sore throat, but the laboratory tests results were normal. In 2013, a multiple allergen simultaneous test (MAST) was performed; the result was non-specific except for a weak positive outcome for house dust mites. There was no improvement in the symptoms, so she was treated for rheumatism at the hospital. Even though the treatment was conducted, her symptoms continued to be aggravated by city gas fumes. Hence, she plans to move to a new house where city gas is not used.

### Past history

Atopy, hormonal treatment of menopause symptoms (5 years).

### Family history

There is no family history of skin disease. Her daughter did not complain of symptoms similar to her when her daughter came home to visit.

### Social history

No smoking. No drinking.

### Occupational history

She operated a bar on the second floor above a restaurant. She quit her job 1 year later from first induction of symptoms.

### Exercise history

In the past, she jogged almost every day for two hours. But during the last two years, she has usually exercised by walking.

### Co-worker reports

The bar employees reported being aware of smelling ignition coal, but none experienced symptoms such as swelling, sputum, and myalgia like she did.

Results of one set of hospital tests: A clinical examination including a complete blood count (CBC), liver-renal function test (LRFT), electrolyte tests, rheumatoid factor (RF), uric acid, hepatitis B test, antinuclear antibody (ANA test), anti-cyclic citrullinated peptide antibody (anti-CCP antibody), thyroid-stimulating hormone (TSH), and urine analysis (UA) were performed, 2011. No specific findings were revealed except mild elevation of aspartate aminotransferase (AST) and alkaline phosphatase (ALP) and cholesterol. After that, 10 months later, she underwent a re-examination for CBC, LRFT, uric acid, and electrolytes but the results were within normal range again. Three more months later, pulmonary function tests and simple chest radiography and chest computed tomography did not show specific findings. The test results for MAST were low positive (1+) for total immunoglobulin E and house dust mites (mite-farinae, mite-pterony), and very low positive for Acacia, Timothy grass, dog, and cockroach mix.

Symptom changes before/after exposure to the city gas fumes: The most sensitive triggering substance was city gas. After becoming aware of the smell of city gas, she developed symptoms including dyspnea, chest discomfort, sputum, body swelling, myalgia, arthralgia, and headache within 5 minutes. When she was exposed to the smell of city gas, her dyspnea worsened. Therefore, she had to avoid the place of exposure. When she moved to a different location, her symptoms gradually disappeared within 2–3 hours.

First visit to our hospital: The other hospital examinations showed no abnormal findings from blood tests, urine tests, pulmonary function tests, and lung imaging tests. After medical examination by interview, a physical examination was performed according to her symptoms. We considered MCS, FMS, CFS, and somatization disorder, based on the diagnostic process and differential diagnosis proposed by Chae et al [16].

Physical examination: The patient reported that her body weight was highly changeable after exposure to certain smells. Therefore, we measured her height and weight (148.1 cm, 53 kg). We examined the degree of swelling, but there were no abnormal findings. She also complained of joint pain including that of both metacarpal phalangeal joints (MCP joints), the right metatarsal joint of the thumb, and both the Achilles tendons, but there was no swelling, tenderness, or joint deformity. Although she complained of myalgia (including that of the right arm, left forearm, and both calves), there was no swelling or tenderness.

### The Korean version of the QEESI

**Figure 1:**
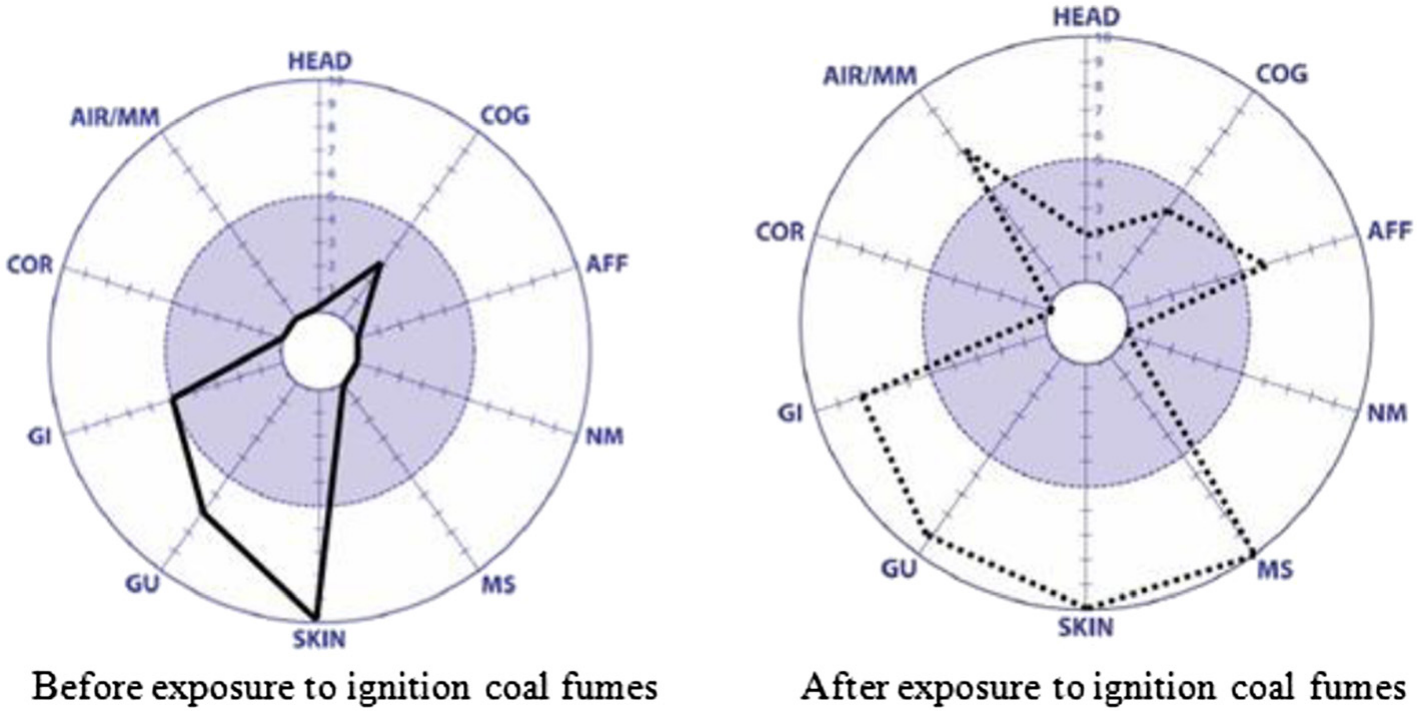
Symptom graph of Korean version of Quick Environmental Exposure and Sensitivity Inventory (QEESI): before and after exposure to ignition coal fumes.

The symptom graph differed before and after exposure to ignition coal (Figure 1). The symptom severity score was 25 points before exposure to ignition coal and it was 56 points after exposure to ignition coal. Before exposure to ignition coal fumes, the score of “skin related symptoms” and “genitourinary related symptoms” was high because of her medical problems with atopic dermatitis and menopause-related symptoms. After exposure to ignition coal fumes, her existing symptoms worsened and new symptoms occurred (including airway or mucous membrane symptoms, musculoskeletal symptoms, affective symptoms, and head-related symptoms). On the Korean Version of the QEESI, all of the area scores were high and very suggestive of MCS.

The symptom severity score was 56 points, chemical intolerance score was 53 points, other intolerance score was 60 points, life impact score was 77 points, and masking index score was 7 points.

Second visit to our hospital, differential diagnosis with FMS: We used the 2010 American College of Rheumatology (ACR) preliminary diagnostic criteria for FMS. We scored each situation, to compare before and after the exposure to the smell. Before exposure to ignition coal, the widespread pain index (WPI) score was 1 point and the symptom severity scale (SSS) score was 8 points. This results did not meet the criteria for a diagnosis of FMS. After exposure to ignition coal, the WPI score was 7 points and the SSS score was 9 points. These met the criteria for the diagnosis of FMS. However, she did not show common FMS clinical features. She had no symptoms of pain worsening with exercise, sleep disturbance (characterized by non-restorative sleep and increased awakenings), or cognitive difficulties including short-term memory problems. After exposure to ignition coal fumes, the diagnostic criteria for FMS was fulfilled because the symptoms are similar to MCS. Hence we excluded FMS.

### Differential diagnosis with CFS

We used the 1994 Centers for Disease Control and Prevention (CDC) diagnostic criteria for CFS. Before exposure to ignition coal fumes, the patient had no symptoms among the diagnostic criteria. However, after exposure to ignition coal, she showed six symptoms among the diagnostic criteria (poor mental function, sore throat, myalgia, multiple arthralgia, new onset headaches, and feeling unwell). However, when she was exposed to fresh air or avoided exposure to triggering substances, all of her symptoms disappeared. Because CFS symptoms are not improved by avoiding the triggers, we excluded CFS.

### Differential diagnosis of somatization disorder

We used the Diagnostic and Statistical Manual of Mental Disorders IV (DSM-IV) in considering somatization disorder. She had social impairment with 4 or more pain symptoms and 2 or more gastrointestinal symptoms, and her symptoms did not start before the age of 30. She also had no sexual symptoms or pseudoneurological symptoms. Therefore, we excluded somatization disorder.

### Second visit to our hospital, physical examination

Her weight was 53.4 kg. She complained of more edema than before. Pitting edema was observed in both forearm regions (1+) and both anterior tibial regions (2+).

### Differential diagnosis of pitting edema

Pitting edema is an abnormal accumulation of fluid in the interstitium characterized by indentations persisting after the release of pressure [17]. Generally, pitting edema occurs because homeostasis of the interstitial fluid has broken. It can be caused by heart failure, liver failure, renal failure, pregnancy, or electrolyte imbalance. She has not observed any organ dysfunction. She was in menopause and her electrolytes were within normal range. No other diseases were suspected.

### Diagnosis of MCS

The patient had multi-organ symptoms when exposed to a triggering chemical that is normally considered to be safe. Her symptoms also occurred repeatedly with chemical exposure, and improved with removal of the chemical exposure. Her symptoms were chronic, and she was sensitive to many chemicals. Considering the diagnostic criteria for MCS agreed upon in Atlanta in 1999 and her results from the Korean version of the QEESI, our case fits the criteria well and the diagnosis is obvious. The patient was educated about MCS and avoiding exposure to triggering substances. She showed changing social behavior in that she moved house twice, reduced her hobbies, and quit her job. Therefore, we aimed to help her return to work and live in her new house by avoiding triggering substances in the environment. She was educated about air purifiers and air purification plants. We also explained long-term care and the necessity of moral support.

## Conclusion

MCS is a term used to describe a disorder characterized by non-specific symptoms, the cause of which is attributed to exposure to extremely low levels of a variety of chemicals. MCS has no consistent diagnostic criteria, and the pathophysiology of MCS is also unknown. Therefore, it is difficult to diagnose MCS. The symptoms are ambiguous and it is difficult to conduct a differential diagnosis from other diseases. Unnecessary tests may be administered because MCS patients have many symptoms. When MCS is suspected, the QEESI [18], which has a Korean-language version, can be used. MCS may be comorbid with allergic diseases. However, allergies alone cannot explain all of its symptoms. In order to diagnose MCS, similar diseases such as somatization disorder, CFS, and FMS should be excluded. However, sometimes MCS may occur with these diseases. A concurrent diagnosis MCS with CFS or FMS can be ambiguous and requires clinical judgment. Because the symptoms of these diseases are very similar, it is difficult to determine whether comorbid diseases exist or the diagnosis is wrong. Since the treatment for these diseases is similar, it is better to treat the symptoms than to do many tests for diagnostic purposes [19]. The treatment of MCS is symptomatic treatment and education on avoiding the chemicals that may trigger symptoms. The most important goal is to enable the patient to return to ordinary life and to work [16].

In this case, pitting edema was observed, but systemic disease was not suspected based on CBC, LRFT, electrolyte tests, and urine analysis. Arthralgia was observed, but rheumatic disease was not suspected. Laboratory test results, including those for RF, uric acid, ANA, and anti-CCP antibodies were within normal range. The MAST and total immunoglobulin E tests had non-specific findings. Hence, the cause of the allergy was not determined. A screening test was performed for all the symptoms of which the patient complained, but the test results produced no specific findings. Hence, the possibility of symptoms caused by a specific physical disease was low. We conducted a differential diagnosis among chronic diseases with non-specific symptoms including MCS, FMS, CFS, and somatization disorder. Before exposure to the ignition coal, she did not fit the 2010 ACR preliminary diagnostic criteria for diagnosis of FMS. However, after exposure to the ignition coal, her symptoms met the criteria for the diagnosis of FMS. This is because of the symptom similarity between FMS and MCS. The patients’ symptoms that were similar to those of FMS would have been caused by MCS. For about 1 year, she had been treated for FMS, but her symptoms had not improved. In addition, her symptoms recurred when she was exposed to certain chemicals. These facts suggested that her symptoms might have been caused by MCS rather than FMS. Following the 1994 CDC diagnostic criteria, CFS symptoms are not improved by rest. However, when exposed to fresh air or upon avoiding exposure to the causal substance, all of her symptoms disappeared. Hence, we excluded CFS. We used the DSM-IV diagnostic criteria for somatization disorder. Her symptoms did not meet the DSM-IV criteria for somatization disorder. We excluded somatization disorder. The Korean version of QEESI was used for a diagnosis of MCS, and the result showed a high possibility of MCS. Most of her symptoms occurred after exposure to chemicals, and the symptoms disappeared when she avoided exposure to those chemicals. The patient was also observed to have changed her behavior: moving house and quitting her job to avoid the fumes. Based on the 1999 consensus diagnosis criteria for MCS, we diagnosed her with MCS.

In this case, ignition coal fumes induced the MCS. Ignition coal is made by forming charcoal powder into briquettes. The charcoal powder is produced by carbonization using wood and sawdust at high temperatures. Hence, ignition coal is a kind of sawdust charcoal. Not only pure wood but also waste wood, waste wooden furniture, and waste plywood are used to make ignition coal. Heavy metals including lead and cadmium [20] as well as VOCs including benzene and toluene [21] have been detected in ignition coal. VOCs are known to cause MCS. Thurs, there is a possibility that the MCS was caused by elements of the ignition coal itself. In western countries, MCS cases caused by environmental exposure such as tobacco, natural gas, and combustion products (i.e. those involving automobile exhaust combustion) have been reported [8,22]. This is similar to our case, which is induced by ignition coal fumes. Ignition coal is a product that reflects Korean food culture, but there is a lack of research on the health effects of ignition coal. As in this case, residents who live nearby restaurants using ignition coal may face health problems after environmental exposure to the ignition coal.

In Korea, MCS cases caused by occupational exposure have been intensively reported. There have been MCS cross-sectional studies (compared with the symptoms of exposure to VOCs) [11,12] and MCS epidemiological studies (self-reported prevalence using the Korean version of the QEESI) [13,14] in Korea. However none but a study on workers in a chemical analysis department followed a differential diagnostic procedure [15]. However, because this study has not been published yet, it is unclear what MCS diagnostic process and differential diagnostic process were used.

For the present case, we conducted various clinical tests to rule out other diseases with similar symptoms. The diagnostic process was described in detail here. This case report should be helpful for diagnosis in cases when MCS is suspected. This is also the first case report on MCS in Korea caused by environmental exposure to everyday materials. The patient initially showed a sensitive response only to the fumes of ignition coal, but she was not diagnosed with MCS. She was diagnosed FMS and treated for her symptoms. Later, she showed hypersensitivity to other chemicals, such as the smell of city gas and a burning smell. Eventually, she suffered from enough pain to quit her job. If occupational and environmental physicians have patients with MCS-like symptoms (such as CFS, FMS, and somatization disorder), considering the possibility of MCS is necessary. If a diagnosis of MCS is made, avoidance of triggering substances is very important in order to prevent symptom expansion.

The patient’s symptoms might include an allergic reaction due to the fumes of chemicals such as ignition coal, city gas, and a burning smell. In order to confirm the diagnosis of allergic disease, an allergic challenge test should be performed. The fact that we did not perform the tests might be a limitation of this report. However, in the patient’s history, the symptoms induced by the exposure to chemicals were obvious. If the challenge test result would have been negative, the challenge test could not have excluded MCS. Hence, we did not perform the challenge test, which would have been a burden to the patient.

We performed a physical examination and clinical examination according to the symptoms of our patient. An MCS screening test was performed using the Korean version of the QEESI. We also conducted a differential diagnostic procedure to exclude diseases similar to MCS such as FMS, CFS, and somatization disorder. We report the first case of MCS due to environmental exposure in Korea in which symptoms first appeared due to the fumes of ignition coal, and sensitivity to other chemicals, such as city gas and burning fumes, developed subsequently.

### Consent

Written informed consent was obtained from the patient for the publication of this report and any accompanying images.

## Competing interests

The authors declare that they have no competing interests.

## Authors’ contributions

YMJ and KDM conceived and designed the study. KYK, KJE and LKH were involved in conduction of the study. YMJ, KDM and LKH were involved in writing the manuscript. YMJ, KDM and LKH performed the revision the manuscript. All authors read and approved the final manuscript.
